# PTEN Reduces BMP9-Induced Osteogenic Differentiation Through Inhibiting Wnt10b in Mesenchymal Stem Cells

**DOI:** 10.3389/fcell.2020.608544

**Published:** 2021-02-04

**Authors:** Fu-Shu Li, Pei-Pei Li, Ling Li, Yan Deng, Ying Hu, Bai-Cheng He

**Affiliations:** ^1^Department of Pharmacology, School of Pharmacy, Chongqing Medical University, Chongqing, China; ^2^Key Laboratory of Biochemistry and Molecular Pharmacology of Chongqing, Chongqing Medical University, Chongqing, China

**Keywords:** phosphatase and tensin homolog deleted on chromosome 10, WNT10B, BMP9, osteogenic differentiation, mesenchymal stem cell

## Abstract

Bone morphogenetic protein 9 (BMP9) is one of the most efficacious osteogenic cytokines. PTEN and Wnt10b are both implicated in regulating the osteogenic potential of BMP9, but the potential relationship between them is unknown. In this study, we determined whether PTEN could reduce the expression of Wnt10b during the osteogenic process initialized by BMP9 in mesenchymal stem cells (MSCs) and the possible molecular mechanism. We find that PTEN is inhibited by BMP9 in MSCs, but Wnt10b is increased simultaneously. The BMP9-induced osteogenic markers are reduced by PTEN but increased by silencing PTEN. The effects of knockdown PTEN on elevating BMP9-induced osteogenic markers are almost abolished by knockdown of Wnt10b. On the contrary, the BMP9-increased ALP activities and mineralization are both inhibited by PTEN but almost reversed by the combination of Wnt10b. Bone masses induced by BMP9 are enhanced by knockdown of PTEN, which is reduced by knockdown of Wnt10b. The BMP9-increased Wnt10b is decreased by PTEN but enhanced by knockdown of PTEN. Meanwhile, the BMP9-induced Wnt10b is also reduced by a PI3K-specific inhibitor (Ly294002) or rapamycin, respectively. The BMP9-induced phosphorylation of CREB or Smad1/5/9 is also reduced by PTEN, but enhanced by PTEN knockdown. In addition, p-CREB interacts with p-Smad1/5/9 in MSCs, and p-CREB or p-Smad1/5/9 are both enriched at the promoter region of Wnt10b. Our findings indicate that inhibitory effects of PTEN on BMP9's osteogenic potential may be partially mediated through decreasing the expression of Wnt10b via the disturbance of interaction between CREB and BMP/Smad signaling.

## Introduction

As a member of the bone morphogenetic protein (BMP) family, BMP9 exhibits more efficacious osteogenic potential than that of BMP2 and/or BMP7 in mesenchymal stem cells (MSCs) (Kang et al., 2004; McKie et al., 2014; Xie and Bu, 2019). It initializes the osteogenic differentiation through BMP/Smad and/or noncanonical BMP/Smad signaling, such as p38 MAPK and PI3K/Akt (Xie and Bu, 2019). Besides this, other diverse factors or signaling pathways also are involved in regulating this biological process, such as the Wnt/β-catenin pathway. BMP9 promotes the activation of Wnt/β-catenin signaling when it commits progenitor cells to osteoblastic lineages, and the BMP9-induced osteogenic process is inhibited substantially by silencing β-catenin (Tang et al., 2009). However, the concrete mechanism about how BMP9 activates Wnt/β-catenin signaling is yet unclear.

The Wnt signaling pathway is very complex and can be classified as the canonical Wnt (also called Wnt/β-catenin) and noncanonical Wnt pathways. It regulates cell proliferation, differentiation, survival, and other physiological processes (Wiese et al., 2018). In development, the Wnt/β-catenin signaling pathway is necessary to create homeostasis in the skeletal system (Girardi and Le Grand, 2018). Wnt10b is a secreted protein, which can specifically activate Wnt/β-catenin signaling (Wright et al., 2007). Wnt10b may be associated with regulating bone mass and maintaining the progenitor activity of bone marrow stem cells (Stevens et al., 2010). Besides this, it may function as a switcher between adipogenesis and osteogenesis. Thus, the effect of Wnt10b on promoting bone formation may due to the expense of adipogenesis (Cawthorn et al., 2012). The bone mass and mechanical properties were elevated in Wnt10b transgenic mice, and trabecular bone was decreased if Wnt10b was knocked out (Bennett et al., 2005). Our previous study demonstrates that BMP9 upregulates Wnt10b greatly in MSCs, and the osteogenic potential of BMP9 is decreased obviously by silencing Wnt10b (Liao et al., 2019). Hence, upregulation of Wnt10b may be one of the essential mechanisms about how BMP9 activates Wnt/β-catenin signaling in MSCs. However, the molecular mechanism about how BMP9 promotes the expression of Wnt10b remains unclear.

PTEN is a well-known tumor suppressor, which can negatively regulate the PI3K/Atk pathway to inhibit proliferation and survival of cancer cells (Georgescu, 2010). Apart from cancer, PTEN is also involved in regulating skeletal development. Bone mass density is increased progressively in PTEN-deficient mice (Liu et al., 2007). Our previous study indicates that the osteogenic potential of BMP9 is enhanced by silencing PTEN (Huang et al., 2014). As reported, PTEN can reduce the activation of the Wnt/β-catenin signaling pathway through regulating the phosphorylation of GSK3β (Persad et al., 2016). Thus, PTEN is also involved in mediating the effect of BMP9 on enhancing Wnt/β-catenin signaling in MSCs. However, it remains unknown whether PTEN could modulate the activation of Wnt/β-catenin signaling through regulating the expression of Wnt10b.

In this study, we determined whether Wnt10b could reverse the inhibitory effect of PTEN on the BMP9-induced osteogenic process in MSCs and dissect the possible relationship between PTEN and Wnt10b during the osteoblastic commitment initialized by BMP9 in progenitor cells.

## Materials and Methods

### Cell Culture and Chemicals

C3H10T1/2 cells were bought from ATCC (Manassas, VA, USA) and cultured with complete Dulbecco's modified Eagle's medium, which containing 10% fetal bovine serum (FBS), 100 μg/ml streptomycin, and 100 U/ml penicillin. Besides this, cells were saturated at 37°C and 5% CO_2_. Primary antibodies against PTEN (sc-7974), Runx2 (sc-390715), OPN (sc-21742), and GAPDH (sc-47724) were ordered from Santa Cruz Biotechnology; Wnt10b (ab70816), CREB (ab32515), and p-CREB (ab32096) were ordered from Abcam; Smad1/5/9 (13820S) was ordered from CST; and p-Smad1/5/9 (AF8313) was bought from Affinity Biotech.

### Recombinant Adenoviral Construction

Recombinant adenoviruses used for this study were constructed following the AdEasy system (He et al., 1998; Luo et al., 2007). Briefly, the coding sequence of mouse PTEN, BMP9, and Wnt10b were amplified with PCR. The PCR products as well as the siRNA oligo fragments for PTEN or Wnt10b were all subcloned into adenoviral shuttle vectors, respectively. Then, we linearized these vectors and recombined them homologously in BJ/5183 cells. Finally, recombinant viruses were packaged in HEK293 cells (from ATCC), and products were designated as AdPTEN, AdWnt10b, AdBMP9, AdsiPTEN, and AdsiWnt10b. These adenoviruses were all tagged with GFP for tracking the virus, and the adenovirus expressing GFP only (AdGFP) was used as a vector control.

### Reverse Transcription (RT) Reaction and Quantitative Polymerase Chain Reaction (PCR) Assay

Total RNAs were extracted from cells with TRIzol, and the cDNAs were produced with the RT kit (Cat. No. R037A, Takara). SYBR green mixture kits were used for real-time PCR assay with the Bio-Rad CFX Connect system. The target mRNA levels were normalized with glyceraldehyde phosphate dehydrogenase (GAPDH). Primer sequences used for this study are listed in Table 1.

**Table 1:** The primers used for PCR.

**Gene**	**Primer**	**Sequence (5^′^ → 3^′^)**
Wnt10b	F	GGATGGAAGGGTAGTGGTGA
	R	CTCTCCGAAGTCCATGTCGT
Runx2	F	GCCGGGAATGATGAGAACTA
	R	GGACCGTCCACTGTCACTTT
PTEN	F	CATAACGATGGCTGTGGTTG
	R	CGGGGTAAGGCTGTTTTACA
GAPDH	F	ACCCAGAAGACTGTGGATGG
	R	CACATTGGGGGTAGGAACAC
Wnt10b (ChIP)	Primer1 F	AGCTAGGAGGGTGAGTCAGG
	Primer1 R	TGCTGCACAAGAGATGAGGG
	Primer2 F	GCTGGCCCATCTCAGAAGTT
	Primer2 R	GCTTCCTTGATGAGGGTGCT
	Primer3 F	GTCCTCAGCGTGTCAAAGGA
	Primer3 R	GAGTTCCACTCACCTGCTCC

*F, forward; R, reverse*.

### Protein Harvest and Western Blot Assay

At the end point, culture medium was discarded, and the cells were washed twice with ddH_2_O. Then, 300 μl RIAP lysis solution (R0020, Solarbio, China) was added to each well, and we collected the lysates that were denatured by boiling for 10 min. Protein samples were resolved with 10% SDS-PAGE gel. Then, proteins were transferred to a polyvinylidene difluoride membrane and subjected to the standard Western blot analysis. Finally, the target proteins were developed with a chemiluminescent substrate kit (160072, Saimike biotech, Chongqing China). The data were collected and subjected to quantitative analysis with the Bio-Rad Chemx system (Bio-Rad, USA).

### Alkaline Phosphatase (ALP) Activities Assay

ALP activities were determined on days 5 and 7. Briefly, cells were plated in 24-well-plates and treated according to the experimental design. At each time point, cells were subjected to staining with NBT/BCIP kits (C3206, Beyotime, China) following the manufacturer's instructions.

### Matrix Mineralization Assay

The mineralized matrix nodules were visualized with Alizarin Red S (A5533-25G, Sigma-Aldrich). Briefly, cells were seeded in 24-well-culture plates and treated according to the experimental design. On day 20 after being treated, the mineralized nodules were stained with 0.4% Alizarin Red S as described previously (Wang et al., 2019). Each assay was repeated in triplicate independently.

### Stem Cell Transplantation Assay

Cells were pretreated according to the experimental design. Twenty-four hours later, cells were collected, resuspended in PBS (4°C), and implanted subcutaneously to the flank of nude mice (five per group, females, 4–6 weeks, 20–24 g body weight) by injection (5 × 10^6^ cells per injection). The mice were ordered from the animal center of Chongqing Medical University (Chongqing, China). The present experiment was approved by the institutional animal care and use committee of Chongqing Medical University (Chongqing, China). Five weeks after transplantation, animals were euthanized, and bone masses were retrieved for the following image and histological analysis.

### Micro-Computed Tomographic (μ-CT) Assay

Samples were scanned with the VivaCT 40 μ-CT system (SCANCO Medical AG, Switzerland). The 3-D reconstruction and quantitative analysis were conducted with μ-CT 516.1 (provided by the scanner manufacturer).

### Histological Evaluation

Bone masses were fixed with 10% formalin, decalcified, and then embedded in paraffin. The sections were subjected to hematoxylin and eosin (H&E) staining after being deparaffinized and rehydrated.

### Chromatin Immunoprecipitation Assay (ChIP)

Cells were seeded in T75 flasks and pretreated with AdBMP9. Thirty hours after being treating, cells were cross-linked. The samples were subjected to the standard ChIP analysis procedure as described previously (Tang et al., 2009). The primary antibodies against p-Smad1/5/9, p-CREB, or rabbit IgG were used to pull down the protein and DNA complex. The enrichment of Wnt10b promoter fragments were detected with PCR, and the specific primer sequences are presented in Table 1.

### Immunoprecipitation Assay

Cells were seeded in 6-well-plates and treated according to the experimental design. After 30 h, cells were washed with PBS (4°C) and treated with RIPA lysis buffer on ice, which contained protease and phosphotase inhibitors. Protein G magnetic beads were pretreated with RIPA lysis buffer, and the lysates were pretreated with these beads. Then, the lysate products were incubated at 4°C overnight with antibodies against p-Smad1/5/9, p-CREB, or rabbit IgG and then followed by incubating with protein G magnetic beads. The precipitants were washed with RIPA lysis buffer carefully, and proteins were eluted from beads with SDS sample buffer. Finally, the protein samples were subjected to standard Western blot assay.

### Statistical Analysis

The data were presented as mean ± SD and analyzed with GraphPad Prism 6. Two-tailed Student's *t*-test or one-way analysis of variance with Tukey's *post-hoc* test were used to evaluated the differences. Statistical significance was defined if the value of *p* is < 0.05.

## Results

### Effects of BMP9 on PTEN and Wnt10b in Multiple Progenitor Cells

**Figure 1:**
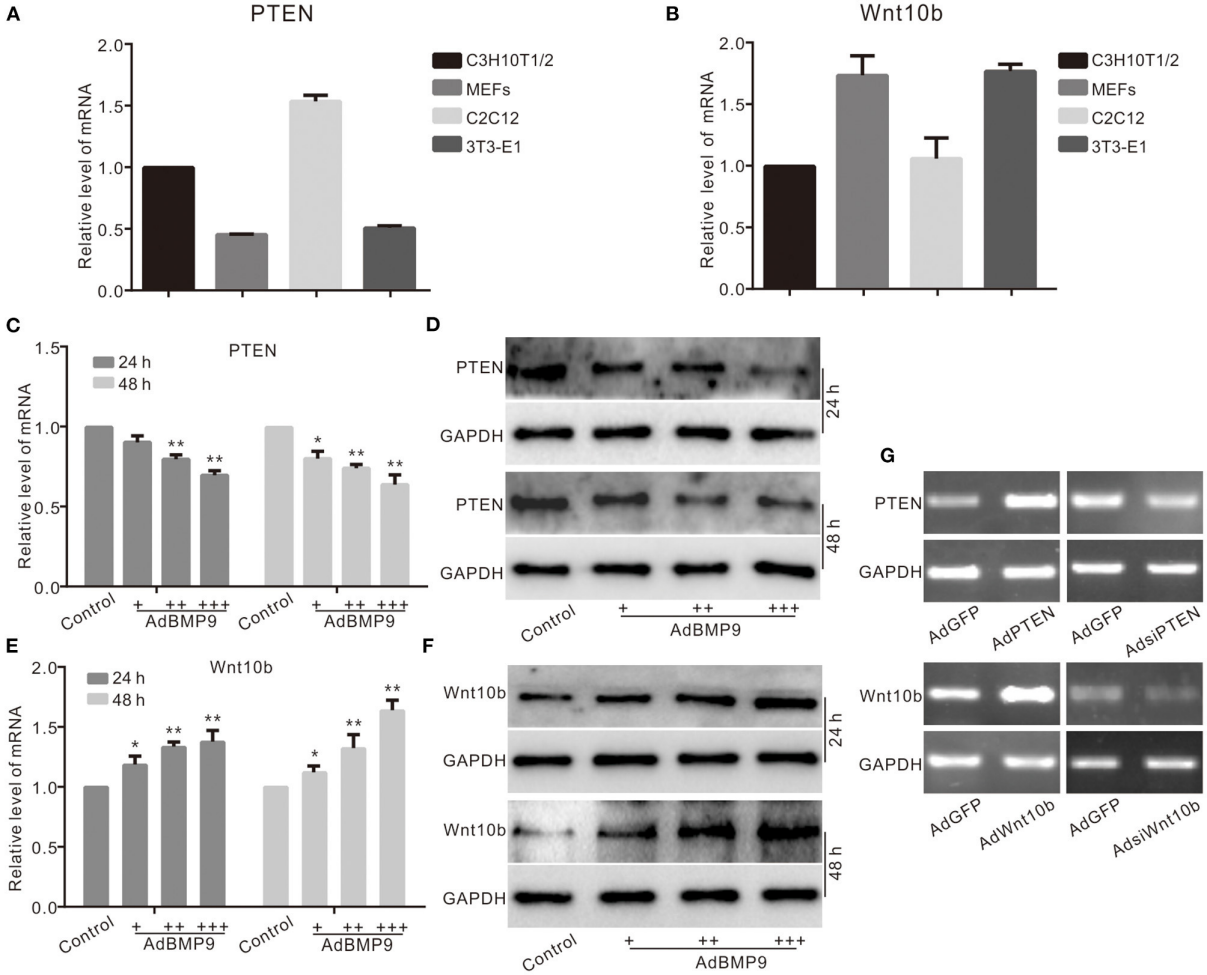
Effects of BMP9 on PTEN and Wnt10b in multiple progenitor cells. **(A)** Real-time PCR assay results show the endogenous mRNA expression of PTEN in different progenitor cells. **(B)** Real-time PCR assay results show the endogenous mRNA expression of Wnt10b in these progenitor cells. **(C)** Real-time PCR assay results show the effect of BMP9 on mRNA expression of PTEN in C3H10T1/2 cells (*p < 0.05, **p < 0.01 vs. control). **(D)** Western blot assay results show the effect of BMP9 on PTEN in C3H10T1/2 cells. **(E)** Real-time PCR assay results show the effect of BMP9 on Wnt10b in C3H10T1/2 cells (*p < 0.05, **p < 0.01 vs. control). **(F)** Western blot assay results show the effect of BMP9 on Wnt10b in C3H10T1/2 cells. **(G)** PCR assay results show the effect of recombinant adenoviruses on the mRNA level of PTEN or Wnt10b in C3H10T1/2 cells.

PTEN or Wnt10b was involved in the osteogenic differentiation induced by BMP9 in multiple progenitor cells, but the relationship between them is unknown. Real-time PCR results show that PTEN and Wnt10b are both detectable in several progenitor cells (Figures 1A,B). BMP9 decreased the mRNA and protein level of PTEN in C3H10T1/2 cells (Figures 1C,D). On the contrary, the mRNA and protein levels of Wnt10b were upregulated by BMP9 in C3H10T1/2 cells (Figures 1E,F). PCR results show the recombinant adenovirus vectors increase or decrease the mRNA level of PTEN or Wnt10b in C3H10T1/2 cells (Figure 1G). These results suggest that PTEN may coordinate to regulate the osteogenic potential of BMP9 in MSCs.

### Effects of PTEN on BMP9-Induced Osteogenic Markers in C3H10T1/2 Cells

**Figure 2:**
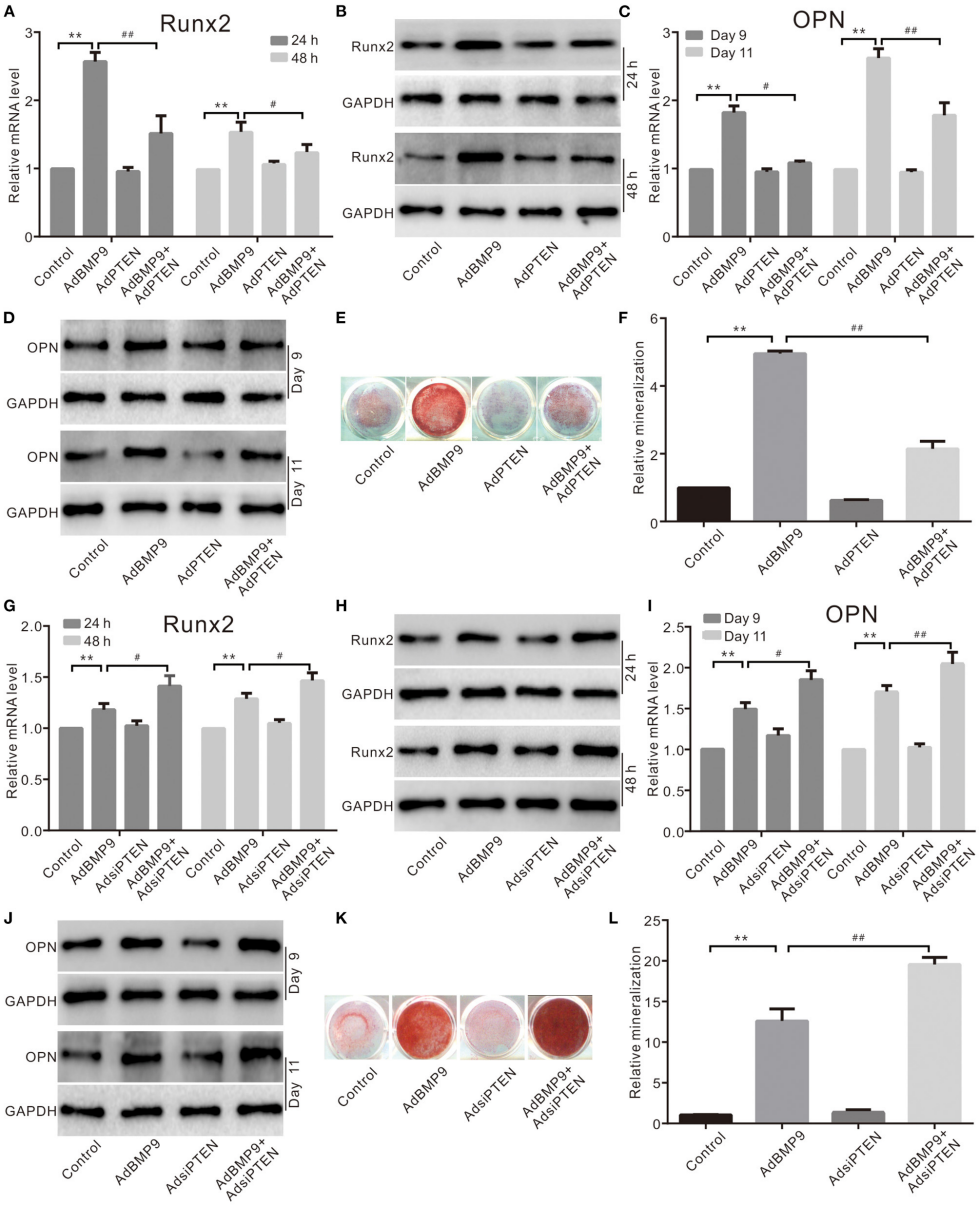
Effects of PTEN on BMP9-induced osteogenic markers in C3H10T1/2 cells. **(A)** Real-time PCR assay shows the effect of PTEN and/or BMP9 on the mRNA expression of Runx2 in C3H10T1/2 cells (**p < 0.01 vs. control; ^#^*p* < 0.05 and ^##^*p* < 0.01). **(B)** Western blot assay shows the effect of PTEN and/or BMP9 on Runx2 in C3H10T1/2 cells. **(C)** Real-time PCR assay shows the effect of PTEN and/or BMP9 on the mRNA expression of OPN in C3H10T1/2 cells (**p < 0.01 vs. control; ^#^*p* < 0.05 and ^##^*p* < 0.01). **(D)** Western blot assay shows the effect of PTEN and/or BMP9 on OPN in C3H10T1/2 cells. **(E)** Alizarin Red S staining shows the effect of PTEN and/or BMP9 on mineralization in C3H10T1/2 cells. **(F)** Quantification of Alizarin Red S staining shows the effect of PTEN and/or BMP9 on mineralization in C3H10T1/2 cells (**p < 0.01 vs. control; ^##^*p* < 0.01). **(G)** Real-time PCR assay shows the effect of BMP9 and/or PTEN knockdown on Runx2 in C3H10T1/2 cells (**p < 0.01 vs. control; ^##^*p* < 0.01). **(H)** Western blot assay shows the effect of BMP9 and/or PTEN knockdown on Runx2 in C3H10T1/2 cells. **(I)** Real-time PCR assay shows the effect of BMP9 and/or PTEN knockdown on OPN in C3H10T1/2 cells (**p < 0.01 vs. control; ^#^*p* < 0.05 and ^##^*p* < 0.01). **(J)** Western blot assay shows the effect of BMP9 and/or PTEN knockdown on OPN in C3H10T1/2 cells. **(K)** Alizarin Red S staining shows the effect of BMP9 and/or PTEN knockdown on mineralization in C3H10T1/2 cells. **(L)** Quantification results of Alizarin Red S staining show the effect of BMP9 and/or PTEN knockdown on mineralization in C3H10T1/2 cells (**p < 0.01 vs. control; ^##^*p* < 0.01).

To determine the possible relationship between PTEN and Wnt10b in the BMP9-induced osteoblastic differentiation, we recapitulated the effect of PTEN on the osteogenic potential of BMP9. The mRNA and protein levels of Runx2 induced by BMP9 were decreased by PTEN in C3H10T1/2 cells (Figures 2A,B). The mRNA and protein levels of OPN were increased by BMP9 but decreased by the combination of PTEN in C3H10T1/2 cells (Figures 2C,D). Meanwhile, the BMP9-induced mineralization was also inhibited by PTEN (Figures 2E,F). On the contrary, knockdown of PTEN potentiated the effects of BMP9 on Runx2 (Figures 2G,H), OPN (Figures 2I,J), and mineralization (Figures 2K,L). These results suggest that the osteogenic potential of BMP9 can be inhibited by PTEN in MSCs.

### Effects of Wnt10b and/or PTEN on BMP9-Induced Osteogenic Markers in C3H10T1/2 Cells

**Figure 3:**
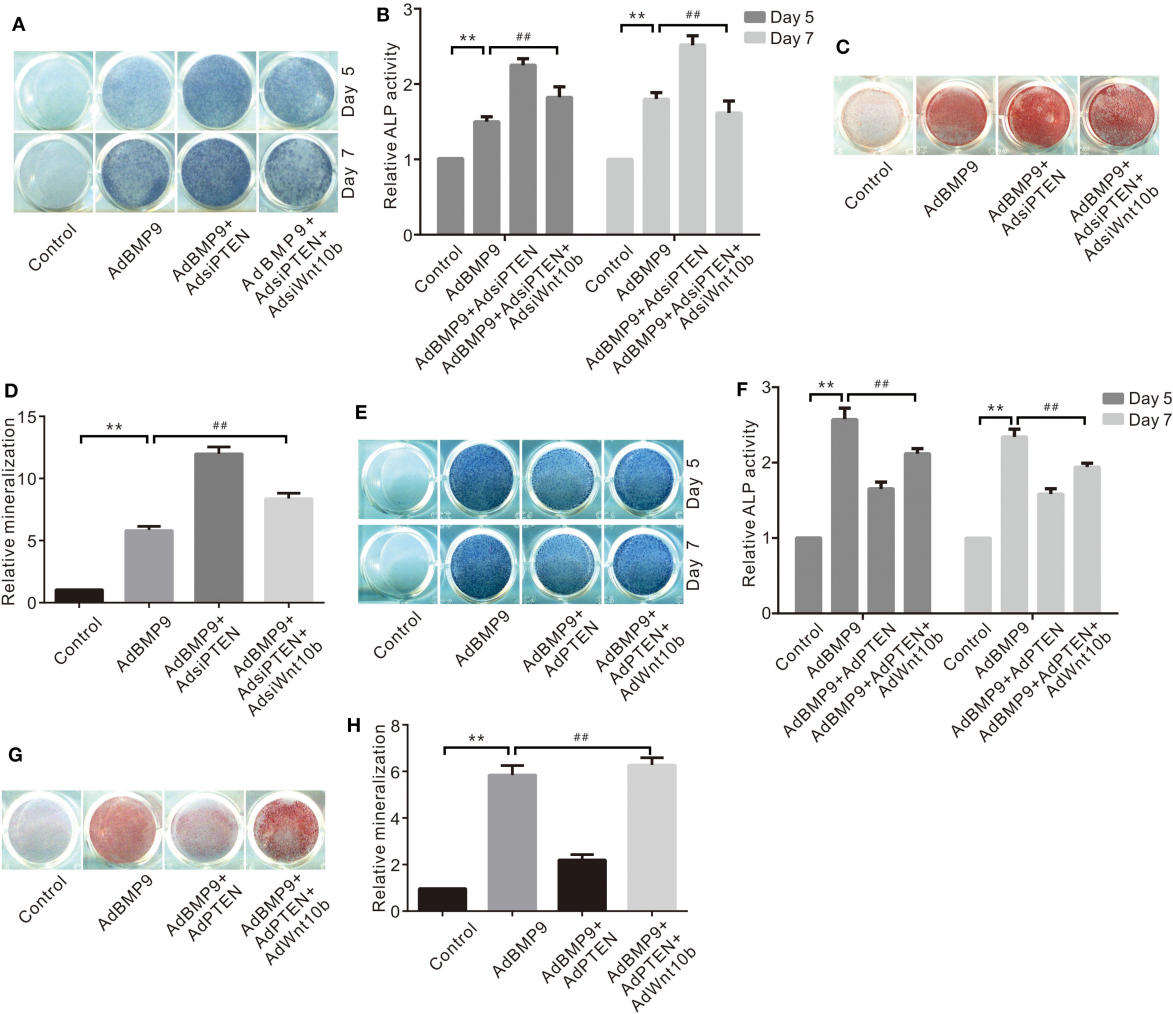
Effects of Wnt10b and/or PTEN on BMP9-Induced Osteogenic Markers in C3H10T1/2 Cells. **(A)** Histochemical staining results show the effect of PTEN knockdown and/or Wnt10b knockdown on ALP activities induced by BMP9. **(B)** Quantification results of histochemical staining show the effect of PTEN knockdown and/or Wnt10b knockdown on ALP activities induced by BMP9 (**p < 0.01 vs. control, ^##^*p* < 0.01). **(C)** Alizarin Red S staining results show effects of PTEN knockdown and/or Wnt10b knockdown on ALP activities induced by BMP9. **(D)** Quantification results of Alizarin Red S staining show effects of PTEN knockdown and/or Wnt10b knockdown on ALP activities induced by BMP9 (**p < 0.01 vs. control, ^##^*p* < 0.01). **(E)** Histochemical staining results show the effect of PTEN and/or Wnt10b on ALP activities induced by BMP9. **(F)** Quantification results of histochemical staining show the effect of PTEN and/or Wnt10b on ALP activities induced by BMP9 (**p < 0.01 vs. control, ^##^*p* < 0.01). **(G)** Alizarin Red S staining results show effects of PTEN and/or Wnt10b on ALP activities induced by BMP9. **(H)** Quantification results of Alizarin Red S staining show effects of PTEN and/or Wnt10b on ALP activities induced by BMP9 (**p < 0.01 vs. control, ^##^*p* < 0.01).

Because BMP9 inhibited PTEN and increased Wnt10b simultaneously, Wnt10b may be implicated in the suppressive effects of PTEN on the osteogenic potential of BMP9. ALP activity assay results show that knockdown of PTEN enhances the BMP9–induced ALP activities in C3H10T1/2 cells, but this effect was almost eliminated by silencing Wnt10b (Figures 3A,B). Similarly, knockdown of PTEN potentiated the BMP9-induced mineralization, which was greatly reduced by silencing Wnt10b (Figures 3C,D). On the contrary, PTEN reduced the BMP9-induced ALP activity, which was partly reversed by exogenous Wnt10b in C3H10T1/2 cells (Figures 3E,F). Besides this, PTEN reduced the BMP9-induced mineralization, but it was obviously reversed by Wnt10b (Figures 3G,H). These results suggest that the inhibitory effect of PTEN on BMP9's osteogenic potential can be partially reversed by Wnt10b in MSCs at least.

### Effects of Wnt10b and PTEN on BMP9-Induced Osteogenesis in C3H10T1/2 Cells

**Figure 4:**
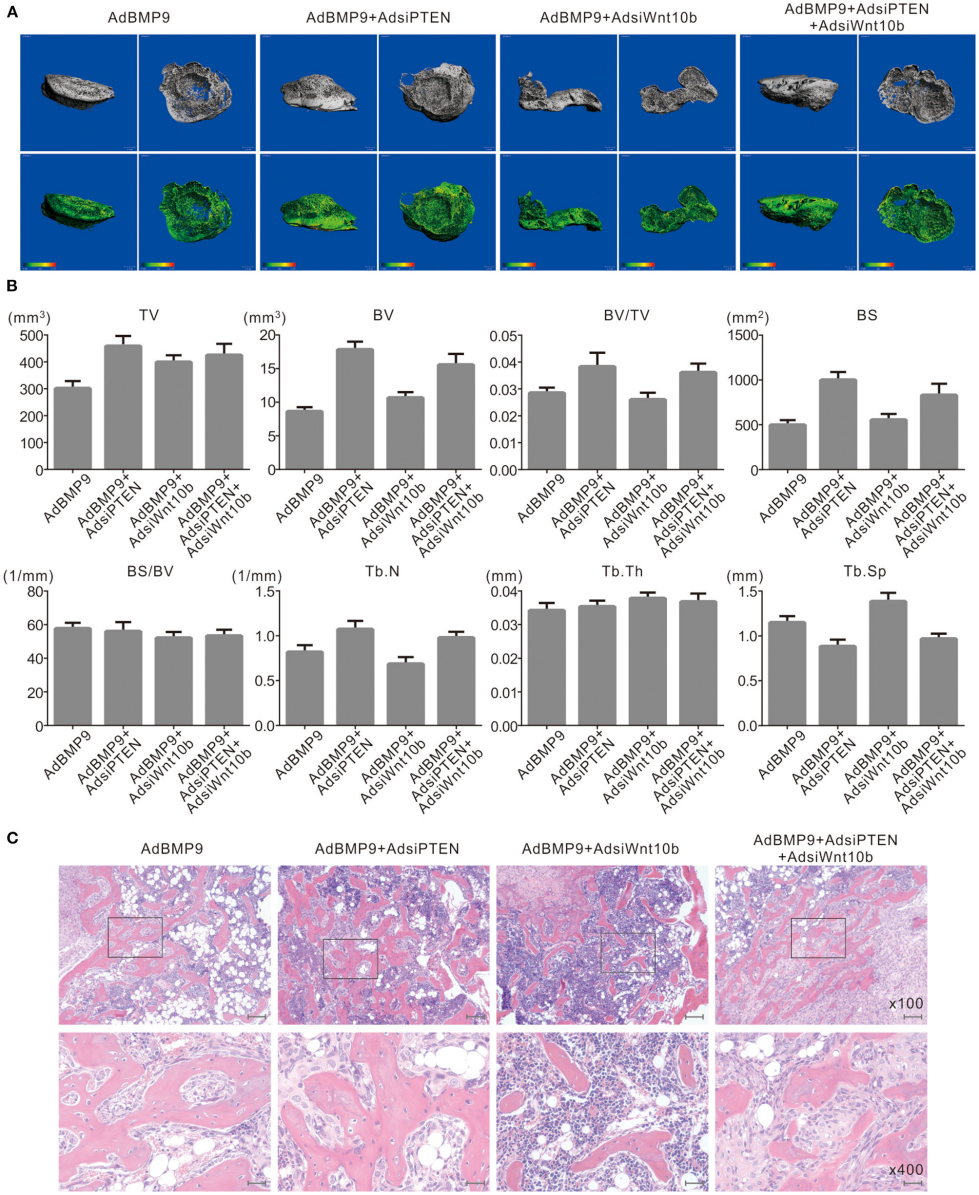
Effects of Wnt10b and PTEN on BMP9-induced osteogenesis in C3H10T1/2 cells. **(A)** 3-D reconstruction of μ-CT scanning results shows the effect of silencing Wnt10b and/or PTEN on BMP9-induced bone formation in C3H10T1/2 cells (representative data are shown). **(B)** Quantitative analysis μ-CT scanning results show the effect of silencing Wnt10b and/or silencing PTEN on BMP9-induced bone formation in C3H10T1/2 cells (TV, total volume; BV, bone volume; BS, bone surface area; Tb.N, trabecular number; Tb.Th, trabecular thickness; Tb.Sp, trabecular separation). **(C)** H and E staining results show the effect of silencing Wnt10b and/or PTEN on BMP9-induced bone formation in C3H10T1/2 cells (Scale is 200 μm for upper panel; scale bar is 50 μm for lower panel).

Next, we employed a stem cell implantation assay to evaluate the possible relationship between PTEN and Wnt10b in the osteogenesis initialized by BMP9. The 3-D reconstruction of μ-CT scanning results show that knockdown of PTEN increases the bone volume and trabecular number compared with the group treated with BMP9 only, and the bone volume and trabecular number induced by BMP9 were decreased by silencing Wnt10b. However, knockdown of Wnt10b almost abolished the effect of PTEN knockdown on promoting BMP9-induced bone formation (Figures 4A,B). H&E staining results also show that knockdown of PTEN potentiated the effect of BMP9 on increasing trabecular bone, and knockdown of Wnt10b exhibited a reversal effect and almost diminished the effect of PTEN knockdown on enhancing BMP9-induced bone formation (Figure 4C). These data suggest that the negative effect of PTEN on the osteogenic function of BMP9 may be reversed by Wnt10b in MSCs.

### Effects of PTEN, PI3K, or mTOR on BMP9-Induced Wnt10b in C3H10T1/2 Cells

**Figure 5:**
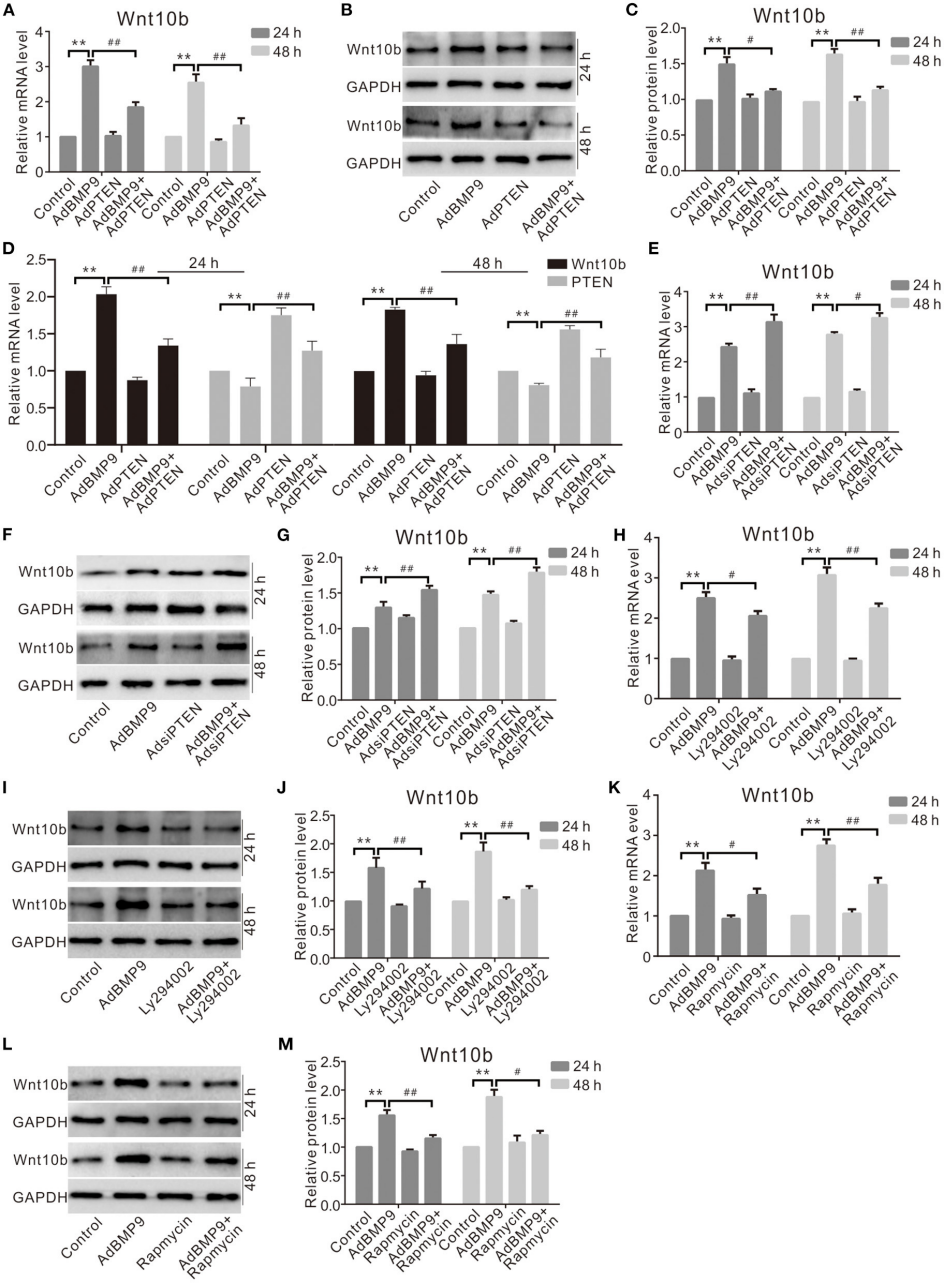
Effects of PTEN, PI3K, or mTOR on Wnt10b induced by BMP9 in C3H10T1/2 cells. **(A)** Real-time PCR assay shows the effect of PTEN and/or BMP9 on Wnt10b in C3H10T1/2 cells (**p < 0.01 vs. control; ^#^*p* < 0.05 and ^##^*p* < 0.01 vs. groups treated with BMP9 only). **(B)** Western blot assay shows effects of PTEN and/or BMP9 on Wnt10b in C3H10T1/2 cells. **(C)** Quantification of Western blot assay shows effect of PTEN and/or BMP9 on Wnt10b in C3H10T1/2 cells (**p < 0.01 vs. control; ^##^*p* < 0.01 vs. groups treated with BMP9 only). **(D)** Real-time PCR results show the effect of exogenous PTEN and/or BMP9 on Wnt10b in C3H10T1/2 cells as well as PTEN (**p < 0.01 vs. control). **(E)** Real-time PCR assay shows the effect of PTEN knockdown and/or BMP9 on Wnt10b in C3H10T1/2 cells (**p < 0.01 vs. control; ^##^*p* < 0.01 vs. groups treated with BMP9 only). **(F)** Western blot assay shows the effect of PTEN knockdown and/or BMP9 on Wnt10b in C3H10T1/2 cells. **(G)** Quantification of Western blot assay shows the effect of PTEN knockdown and/or BMP9 on Wnt10b in C3H10T1/2 cells (**p < 0.01 vs. control; ^#^*p* < 0.05 and ^##^*p* < 0.01 vs. groups treated with BMP9 only). **(H)** Real-time PCR assay shows effects of Ly294002 and/or BMP9 on Wnt10b in C3H10T1/2 cells (**p < 0.01 vs. control; ^##^*p* < 0.01 vs. groups treated with BMP9 only). **(I)** Western blot assay shows the effect of Ly294002 and/or BMP9 on the level of Wnt10b in C3H10T1/2 cells. **(J)** Quantification of Western blot assay shows effects of Ly294002 and/or BMP9 on Wnt10b in C3H10T1/2 cells (**p < 0.01 vs. control; ^##^*p* < 0.01 vs. groups treated with BMP9 only). **(K)** Real-time PCR assay shows the effect of rapamycin and/or BMP9 on Wnt10b in C3H10T1/2 cells (**p < 0.01 vs. control; ^##^*p* < 0.01 vs. groups treated with BMP9 only). **(L)** Western blot assay shows the effect of rapamycin and/or BMP9 on Wnt10b in C3H10T1/2 cells. **(M)** Quantification of Western blot assay shows effect of rapamycin and/or BMP9 on Wnt10b in C3H10T1/2 cells (**p < 0.01 vs. control; ^#^*p* < 0.05 and ^##^*p* < 0.01 vs. groups treated with BMP9 only).

Although Wnt10b may reverse the suppressive effect of PTEN on the osteogenic potential of BMP9, the concrete relationship between them is unclear. Real-time PCR and Western blot assay results show that the effect of BMP9 on upregulating Wnt10b is inhibited by exogenous PTEN (Figures 5A–D), but it is promoted by knockdown of PTEN (Figures 5E–G). Further analysis indicates that the effects of BMP9 on increasing the mRNA and protein levels of Wnt10b were decreased by the specific inhibitor of PI3K/Akt (Figures 5H–J) or rapamycin obviously (Figures 5K–M). These data suggest that PTEN may negatively regulate the expression of Wnt10b in MSCs at least.

### Effects of CREB and BMP/Smad Signaling on the Expression of Wnt10b in C3H10T1/2 Cells

**Figure 6:**
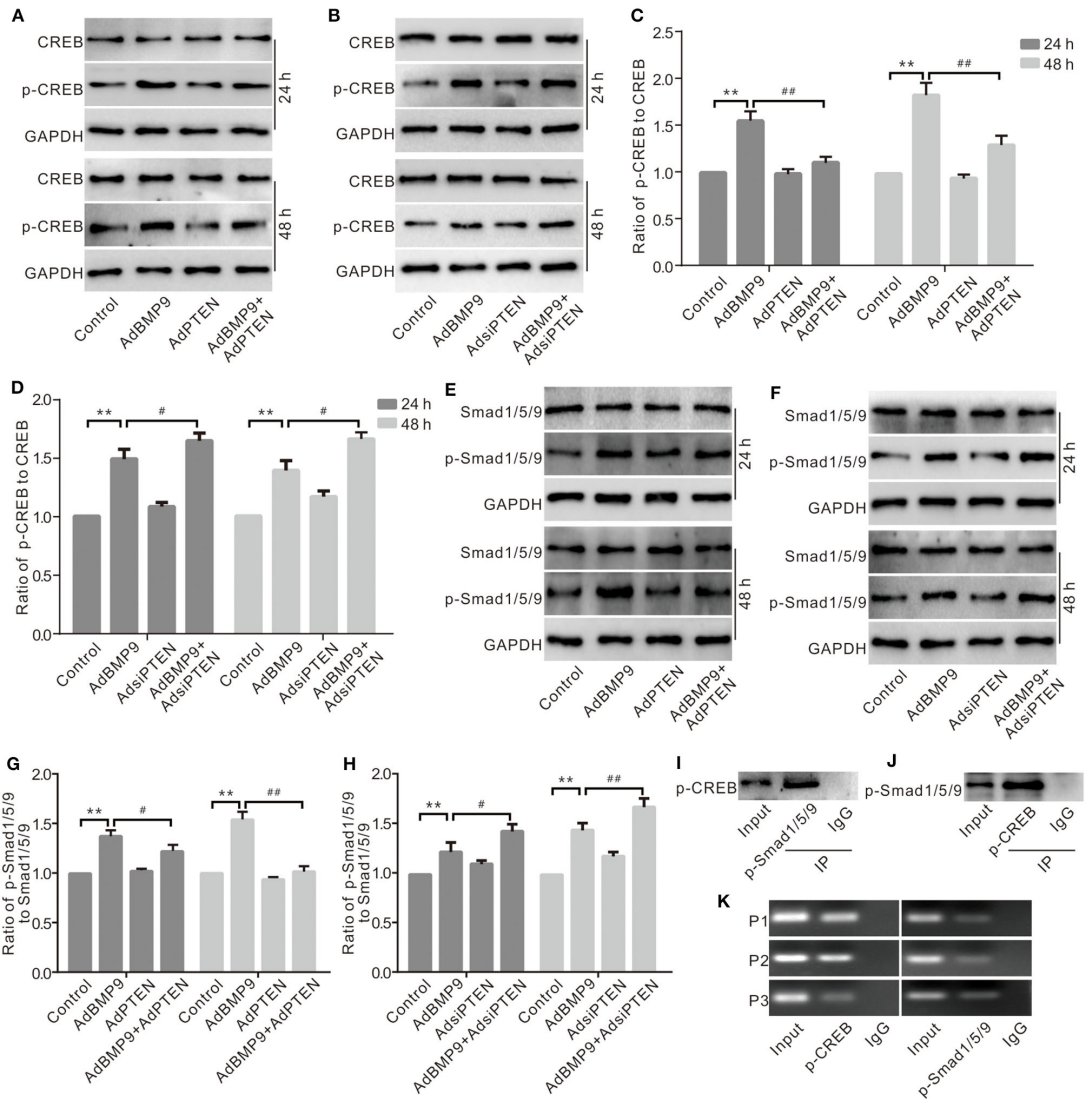
Effects of CREB and BMP/Smad signaling on the expression of Wnt10b in C3H10T1/2 cells. **(A)** Western blot assay shows the effect of PTEN and/or BMP9 on CREB and phospho-CREB (p-CREB) in C3H10T1/2 cells. **(B)** Western blot assay shows the effect of PTEN knockdown and/or BMP9 on CREB and p-CREB in C3H10T1/2 cells. **(C)** Quantification of Western blot assay shows the effect of PTEN and/or BMP9 on p-CREB in C3H10T1/2 cells (**p < 0.01 vs. control; ^#^*p* < 0.05 and ^##^*p* < 0.01 vs. groups treated with BMP9 only). **(D)** Quantification of Western blot assay shows effect of PTEN knockdown and/or BMP9 on p-CREB in C3H10T1/2 cells (**p < 0.01 vs. control; ^#^*p* < 0.05 and ^##^*p* < 0.01 vs. groups treated with BMP9 only). **(E)** Western blot assay shows the effect of PTEN and/or BMP9 on Smad1/5/9 and p-Smad1/5/9 in C3H10T1/2 cells. **(F)** Western blot assay shows the effect of PTEN knockdown and/or BMP9 on Smad1/5/9 and p-Smad1/5/9 in C3H10T1/2 cells. **(G)** Quantification of Western blot assay shows the effect of PTEN and/or BMP9 on p-Smad1/5/9 in C3H10T1/2 cells (**p < 0.01 vs. control; ^##^*p* < 0.01 vs. groups treated with BMP9 only). **(H)** Quantification of Western blot assay shows effect of PTEN knockdown and/or BMP9 on p-Smad1/5/9 in C3H10T1/2 cells (**p < 0.01 vs. control; ^##^*p* < 0.01 vs. groups treated with BMP9 only). **(I)** IP assay shows the interaction between p-Smad1/5/9 and p-CREB in C3H10T1/2 cells. **(J)** IP assay shows the interaction between p-CREB and p-Smad1/5/9 in C3H10T1/2 cells. **(K)** ChIP assay shows the enrichment of p-CREB or p-Smad1/5/9 in the promoter region of Wnt10b C3H10T1/2 cells.

Although PTEN can regulate the expression of Wnt10b in MSCs, the mechanism associating this process remains unclear. Western blot assay results show that BMP9 increased the phosphorylation of CREB (p-CREB), which was reduced by exogenous PTEN and increased by silencing PTEN (Figures 6A–D). BMP9 also increased the phosphorylation of Smad1/5/9 (p-Smad1/5/9), which was reduced by exogenous PTEN and increased by silencing PTEN (Figures 6E–H). IP assay results show that p-CREB interacts with p-Smad1/5/9 in C3H10T1/2 cells (Figures 6I,J). Further ChIP assay results show that p-CREB or p-Smad1/5/9 both were enriched at the promoter region of Wnt10b (Figure 6K). These data suggested that Wnt10b may be regulated by PTEN through the interaction between CREB and BMP/Smad signaling in MSCs.

## Discussion

PTEN and Wnt10b are both implicated in the regulation of BMP9-induced osteogenesis in MSCs, but the possible relationship between them is unknown. In this study, we demonstrate that the inhibitory effect of PTEN on BMP9-induced osteogenic differentiation can be partially reversed by Wnt10b, and the expression of Wnt10b can be inhibited by PTEN through disturbing the interaction between CREB and BMP/Smad signaling at least.

Osteogenesis is a very complex physiological process, and well orchestrated by a variety of signals and/or factors. Both BMPs and Wnt are critical signals for the commitment of progenitor cells to osteoblastic lineage (Cheng et al., 2003; Luu et al., 2007; Kim et al., 2013). The osteogenic potential of BMPs was first found by Urist in 1965 (Grgurevic et al., 2017). To date, it has been demonstrated that BMP9 may be the most efficacious member of the BMP family to initialize osteogenic differentiation (Kang et al., 2004; Zhang et al., 2019). The BMP9-induced osteogenic differentiation is regulated by various signals or factors, such as Wnt/β-catenin signaling, Notch signaling, retinoic acid signaling, and IGF1 (Zhang et al., 2010; Chen et al., 2016; Cui et al., 2019). Wnt/β-catenin is very important for the development of the skeletal system. Zhan and Tang demonstrate that Wnt3a is upregulated by BMP9, and the osteogenic potential of BMP9 is inhibited when the activity of Wnt/β-catenin signaling is reduced in MSCs or stem cells from dental apical papilla. These effects of Wnt/β-catenin may be mediated through upregulating Runx2 directly by β-catenin (Tang et al., 2009; Zhang et al., 2015). Our previous study finds that Wnt10b is also upregulated by BMP9 in MSCs, and the osteogenic capacity of BMP9 is decreased markedly by silencing Wnt10b (Liao et al., 2019). Thus, the cross-talk between BMP9 and Wnt/β-catenin signaling is uncontroversial in MSCs. However, the concrete mechanism through which BMP9 increases the activation of Wnt/β-catenin signaling is unclear.

PTEN is a well-known tumor suppressor, and its function loss or mutation is related to a lot of cancers (Milella et al., 2015; Bazzichetto et al., 2019). Apart from cancers, the physiological function of PTEN also covers the development and metabolism of the skeletal system. Loss of PTEN may result in bone destruction induced by inflammation through promoting osteoclastogenic differentiation in myeloid cells (Bluml et al., 2015). Conditional loss of PTEN in chondrocyte causes kyphosis, a higher adipocyte population in bone marrow, and abnormally fused growth plates in long bones (Hsieh et al., 2009). PTEN loss in osteoblasts dramatically increases bone mineral density throughout life (Liu et al., 2007). All these effects of PTEN on the skeletal system may mostly depend on PI3K/Akt signaling. The activation of PI3K/Akt signaling promotes the phosphorylation of GSK-3β and then increases the level or changes the subcellular location of β-catenin to strengthen the activity of Wnt/β-catenin signaling (Perry et al., 2011). Our previous study shows that PTEN is downregulated by BMP9 during the osteogenic process in MSCs (Huang et al., 2014). Thus, the cross-talk between BMP9 and Wnt/β-catenin signaling may partially result from the BMP9-induced downregulation of PTEN at least.

Wnt/β-catenin signaling can be modulated at different nodes by diversity of factors along the signaling transduction process, such as porcupine, Wif1, DDK1, and sclerostin (Urakami et al., 2006; Baron and Rawadi, 2007; Zhang et al., 2008; Boone et al., 2016). Several skeletal diseases are due to aberrant Wnt/β-catenin signaling, such as tetra-amelia syndrome, autosomal recessive osteoporosis pseudoglioma syndrome, and sclerosteosis (Balemans et al., 2001; Gong et al., 2001; Niemann et al., 2004). Wnt10b, a secretory protein special for Wnt/β-catenin signaling, is also implicated in many diseases or development processes, such as age-related bone loss and decreased fat deposit (Aslanidi et al., 2007; Stevens et al., 2010); it may switch between adipogenesis and osteogenesis (Longo et al., 2004; Aslanidi et al., 2007). Thus, osteogenesis may be enhanced by Wnt10b at the expense of adipogenesis, increasing osteogenic transcriptional factors and suppressing the critical adipogenic factors, such as C/EBPa and PPARγ (Bennett et al., 2005). The FABP4-induced expression of Wnt10b obviously decreases total body fat, including brown and white adipose tissue (Longo et al., 2004). Knockout of Wnt10b increases the age-related trabecular bone loss and reduces the number of bone marrow progenitor cells (Stevens et al., 2010). Our previous study demonstrates that Wnt10b can be induced by BMP9 in MSCs, and the BMP9-induced osteogenic markers are all inhibited by silencing Wnt10b obviously (Liao et al., 2019). Thus, Wnt10b may be very important for maintaining bone density and the number of progenitor cells in adult tissues. Although TNFα and sex hormones may specifically regulate Wnt10b in bone marrow immune cells (Collins et al., 2017), it remains unknown how Wnt10b is regulated when BMP9 initializes osteoblastic commitment in progenitor cells.

In our previous studies, we find that PTEN is inhibited by BMP9, but Wnt10b is increased concurrently (Huang et al., 2014; Liao et al., 2019). COX-2 is identified as a target of Wnt/β-catenin signaling, which can promote BMP9-induced osteogenesis (Wang et al., 2013; Huang et al., 2014). However, it is reported that PTEN can be partially regulated by COX-2 via PGE2 signaling (Li et al., 2011). Meanwhile, PGE2 is one of the master regulators for Wnt/β-catenin signaling (Goessling et al., 2009). Besides this, Liao et al. find that Wnt10b may be partially upregulated by BMP9 through COX-2/CREB signaling (Liao et al., 2019). Thus, we speculate that PTEN may reduce the potential of BMP9 on activating Wnt/β-catenin through inhibiting the expression of Wnt10b in multiple progenitor cells. In this study, we confirmed that BMP9 inhibits PTEN and increases Wnt10b simultaneously in MSCs. BMP9-induced osteogenic markers are inhibited by PTEN, but these effects of PTEN are almost reversed by Wnt10b. On the contrary, the BMP9-induced osteogenic markers are promoted by silencing PTEN, but are reduced apparently by silencing Wnt10b. Thus, the inhibitory effect of PTEN on the osteogenic potential of BMP9 may be partially mediated through inhibiting expression of Wnt10b in MSCs. Further analysis results exhibit that the effect of BMP9 on inducing Wnt10b is inhibited by PTEN while it is potentiated by silencing PTEN. Meanwhile, the effect of BMP9 on inducing Wnt10b is reduced by inhibiting PI3K/Akt or mTOR. Hence, Wnt10b may be negatively regulated by PTEN through PI3K/Akt/mTOR signaling. CREB is a downstream effector of mTOR, which may mediate the effect of PTEN on Wnt10b. However, informatics analysis results show no putative binding site of CREB is present in the promoter region of Wnt10b. IP and ChIP assay results indicate that Smad1/5/9 interacts with CREB, and p-CREB or p-Smad1/5/9 both are enriched at the promoter region of Wnt10b.

Taken together, our findings suggest that the inhibitory effect of PTEN on BMP9-induced osteogenic differentiation may be mediated through reducing the expression of Wnt10b, and PTEN may inhibit Wnt10b by partly disturbing the interaction between CREB and BMP/Smad signaling. Meanwhile, PTEN may modulate the activity of Wnt/β-catenin signaling via a Wnt10b-dependent manner although the concrete process needs to be further unveiled.

## Data Availability Statement

The raw data supporting the conclusions of this article will be made available by the authors, without undue reservation.

## Ethics Statement

The animal study was reviewed and approved by institutional animal care and use committee of Chongqing Medical University.

## Author Contributions

F-SL and B-CH designed the study. F-SL, P-PL, LL, YD, and YH performed the experiments. F-SL and B-CH prepared the manuscript. All authors contributed to the article and approved the submitted version.

## Conflict of Interest

The authors declare that the research was conducted in the absence of any commercial or financial relationships that could be construed as a potential conflict of interest.
